# 91. Establishing Proof of Concept for a Bivalent RSVpreF Subunit Vaccine for Maternal Immunization

**DOI:** 10.1093/ofid/ofac492.016

**Published:** 2022-12-15

**Authors:** Eric A F Simões, Shabir A Madhi, Kimberly J Center, Conrado J Llapur, Jose M Novoa Pizarro, Kena A Swanson, David Radley, Stephanie B McGrory, Emily A Gomme, Daniel A Scott, Kathrin U Jansen, William C Gruber, Alejandra C Gurtman

**Affiliations:** Children's Hospital Colorado, Denver, Colorado; South African Medical Research Council Vaccines and Infectious Diseases Analytics Research Unit and African Leadership in Vaccinology Expertise, Faculty of Health Sciences, University of the Witwatersrand, Parktown, Gauteng, South Africa; Pfizer, Collegeville, Pennsylvania; Hospital del Niño Jesús, San Miguel de Tucuman, Tucuman, Argentina; Hospital Padre Hurtado, Santiago, Region Metropolitana, Chile; Pfizer, Collegeville, Pennsylvania; Pfizer, Collegeville, Pennsylvania; Pfizer, Collegeville, Pennsylvania; Pfizer, Collegeville, Pennsylvania; Pfizer, Collegeville, Pennsylvania; Pfizer, Collegeville, Pennsylvania; Pfizer, Collegeville, Pennsylvania; Pfizer, Collegeville, Pennsylvania

## Abstract

**Background:**

Respiratory syncytial virus (RSV) is a major cause of infant morbidity and mortality worldwide and could be preventable by vaccination in pregnancy.

**Methods:**

We conducted a randomized, placebo-controlled phase 2b trial evaluating safety, immunogenicity, and potential efficacy of a bivalent RSV prefusion F vaccine (RSVpreF) in pregnant women and their infants. Participants were randomized between 24- and 36-weeks’ gestation to receive 120 or 240 µg RSVpreF, with or without aluminum hydroxide, or placebo.

**Results:**

This final analysis includes 579 women and 572 infants in 4 countries (Argentina, Chile, South Africa, and the US); 462 (79.8%) women received RSVpreF. Postvaccination reactions, most commonly injection site pain, fatigue, and myalgia, were generally mild-to-moderate. Adverse events (AEs) in the month following vaccination (maternal) or birth (infant) were mostly anticipated events in pregnancy and the neonatal period, respectively, and were similar between vaccine and placebo groups. No AEs were considered related to vaccination.

For all RSVpreF groups, 50% neutralizing titers for both RSV-A and RSV-B rose sharply by 2 weeks after vaccination. At delivery occurring a mean of ∼8 weeks later, geometric mean titer (GMT) ratios for combined RSV-A/B between vaccine and placebo recipients’ infants were 10.9 to 13.6. Transplacental transfer ratios (all groups) were 1.39 to 1.83. Infant GMTs were higher in infants whose mothers had received RSVpreF versus placebo through 6 months of life; the estimated half-life of infant combined 50% RSV-A/B neutralizing titers was 41 days. Infants of women immunized across the range of assessed gestational ages had similar cord blood titers and transplacental transfer ratios. Observed efficacy (95% CI) against medically attended and severe medically attended infant RSV lower respiratory tract illness (LRTI) through 180 days in an exploratory analysis was 84.7% (21.5%, 97.6%) and 91.5% (-5.6%, 99.8%), respectively.

**Conclusion:**

RSVpreF was well-tolerated in pregnant women, elicited robust neutralizing responses with efficient transplacental transfer, and has the potential to prevent infant RSV LRTI.

**Disclosures:**

**Eric A. F. Simões, MD, M.B., B.S., DCH**, Abbvie: DSMB|Astra Zeneca: Grant/Research Support|Bill and Melinda Gates Foundation: Grant/Research Support|Bill and Melinda Gates Foundation: DSMB|GSK Inc.: DSMB|Johnson and Johnson: Grant/Research Support|Merck Inc: Advisor/Consultant|Merck Inc: Grant/Research Support|Nivavax: Grant/Research Support|Pfizer Inc: Advisor/Consultant|Pfizer Inc: Grant/Research Support|Regeneron: Grant/Research Support|Sanofi Pasteur: Advisor/Consultant **Shabir A. Madhi, MBBCh, FCPaeds, MMed, PhD**, AstraZeneca: Funding to institution for conduct of study **Kimberly J. Center, M.D.**, Pfizer: Employee|Pfizer: Stocks/Bonds **Jose M. Novoa Pizarro, MD**, AstraZeneca: Grant/Research Support|Medimmune: Grant/Research Support|MSD: Grant/Research Support|Pfizer: Grant/Research Support|Regeneron: Grant/Research Support **Kena A. Swanson, Ph.D.**, Pfizer: employee of Pfizer **David Radley, MS**, Pfizer: Employee|Pfizer: Stocks/Bonds **Stephanie B. McGrory, B.S.N.**, Pfizer: Employee|Pfizer: Stocks/Bonds **Emily A. Gomme, Ph.D.**, Pfizer: Stocks/Bonds **Daniel A. Scott, MD**, Pfizer: Employee|Pfizer: Stocks/Bonds **Kathrin U. Jansen, PhD**, Pfizer Inc: Employee|Pfizer Inc: Stocks/Bonds **William C. Gruber, MD**, Pfizer Inc: Employee|Pfizer Inc: Stocks/Bonds **Alejandra C. Gurtman, M.D.**, Pfizer: employee of Pfizer.

